# Cell cycle-dependent binding between Cyclin B1 and Cdk1 revealed by time-resolved fluorescence correlation spectroscopy

**DOI:** 10.1098/rsob.220057

**Published:** 2022-06-29

**Authors:** Martina Barbiero, Luca Cirillo, Sapthaswaran Veerapathiran, Catherine Coates, Camilla Ruffilli, Jonathon Pines

**Affiliations:** Cancer Biology, The Institute of Cancer Research Chester Beatty Laboratories, 237 Fulham Road, London, London SW3 6JB, UK

**Keywords:** cyclin, Cdk1, FCS, cell cycle, complex assembly

## Abstract

Measuring the dynamics with which the regulatory complexes assemble and disassemble is a crucial barrier to our understanding of how the cell cycle is controlled that until now has been difficult to address. This considerable gap in our understanding is due to the difficulty of reconciling biochemical assays with single cell-based techniques, but recent advances in microscopy and gene editing techniques now enable the measurement of the kinetics of protein–protein interaction in living cells. Here, we apply fluorescence correlation spectroscopy and fluorescence cross-correlation spectroscopy to study the dynamics of the cell cycle machinery, beginning with Cyclin B1 and its binding to its partner kinase Cdk1 that together form the major mitotic kinase. Although Cyclin B1 and Cdk1 are known to bind with high affinity, our results reveal that in living cells there is a pool of Cyclin B1 that is not bound to Cdk1. Furthermore, we provide evidence that the affinity of Cyclin B1 for Cdk1 increases during the cell cycle, indicating that the assembly of the complex is a regulated step. Our work lays the groundwork for studying the kinetics of protein complex assembly and disassembly during the cell cycle in living cells.

## Graphical abstract

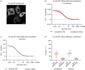

## 1. Introduction

Cell cycle control relies on the rapid formation and disassembly of regulatory protein complexes. At the core of the cell cycle machinery is the family of cyclin-dependent kinases (Cdks; reviewed in [1]) whose members are activated by binding a cyclin subunit. Cyclin B1 binds and activates Cdk1 to form the major mitotic kinase that is required for cells to enter mitosis [2]. Cyclin B1–Cdk1 itself binds to an accessory Cks protein (Cyclin-dependent kinase regulatory subunit Cks1 or Cks2) that recognizes and binds to phospho-threonine ([3–8]; reviewed in [9]). In mitosis, Cyclin B1 binds strongly to the MAD-1 checkpoint protein [10–12], and later to the separase enzyme in a phospho-dependent manner [13–15]. Cyclin B1 levels start to increase late in S phase and continue to accumulate in the cytoplasm of G2 cells [16,17]. Activation of the Cyclin B1–Cdk1-Cks complex sets the time for mitotic entry in all eukaryotes studied to date (reviewed in [18]), whereas its inactivation at metaphase via the ubiquitin-mediated proteolysis of Cyclin B1 is required for cells to exit mitosis [19–21].

At mitosis, the entire cell architecture is reorganized in a matter of minutes. Biochemical analyses have shown that the mitotic kinases and phosphatases have multiple substrates and are often components of several different complexes. Live-cell analyses have shown that cell cycle regulators are often highly dynamic; for example, fluorescence recovery after photobleaching shows that there is a rapid flux of Cyclin B1 on and off the mitotic spindle (H. E. Richardson & J. Pines, unpublished results). Similarly, Förster resonance energy transfer probes specific for different components reveal spatial gradients of activity (reviewed in [22]). Thus, to understand how the cell cycle machinery works, we must measure the kinetics with which regulatory complexes assemble and disassemble, both with respect to cell cycle time and position in the cell.

Biophysical methods such as X-ray crystallography and electron microscopy provide important structural information, and biochemical assays have proved to be invaluable to elucidate the underlying biochemical properties, but because they require populations of lysed cells, they have very limited temporal and spatial resolution, and cannot measure protein dynamics and interactions *in vivo*. By contrast, Fluorescence correlation spectroscopy (FCS) accurately estimates the *in vivo* concentration and dynamics of fluorescently tagged molecules with high spatial and temporal resolution by analysing the intensity fluctuations in a confocal volume (femtolitre scale) [23–29]. Furthermore, fluorescence cross-correlation spectroscopy (FCCS) can measure the interaction between two biomolecules tagged with spectrally distinct fluorophores ([30,31]; reviewed in [23]). Until recently, two factors have limited the application of FCS and FCCS in living cells: the difficulty of expressing a fluorescently labelled protein at a physiologically relevant concentration (i.e. not by overexpression), and the presence of the unlabelled version of the same protein in the cell. The advent of CRISPR/Cas9 gene editing now enables a fluorescent tag to be incorporated into both alleles of a gene to produce a uniformly labelled protein population (reviewed in [32]). Thus, a combination of FCS imaging with CRISPR/Cas9-mediated gene editing offers a platform to study the rapid dynamics of protein complex assembly and disassembly *in vivo*.

In this work, we use CRISPR/Cas9 to tag Cyclin B1 biallelically with the fluorescent protein mEmerald [33] in untransformed human retinal pigment epithelial cells (RPE-1–hTERT; hereafter referred to as RPE-1), to perform FCS and FCCS measurements through the cell cycle and to analyse the dynamics of its assembly into active complexes. Our FCS analysis reveals the existence of two species of Cyclin B1 of different molecular sizes, consistent with a population of free Cyclin B1 and a population of Cyclin B1 bound to its interacting kinase Cdk1. We have validated these results by immunodepletion of Cdk1 in RPE-1 lysates, which confirms the existence of a pool of Cyclin B1 not bound to Cdk1. FCS and FCCS measurements reveal that the fraction of Cyclin B1 bound to Cdk1 increases as cells progress through G2 phase and this is explained by an increase in the affinity of binding. We conclude that the binding between Cyclin B1 and Cdk1 is cell cycle regulated. Overall, our results demonstrate that FCS and FCCS can be used to measure the concentration and interactions of cell cycle proteins in living cells in a time-resolved manner, which will increase our understanding of how the cell cycle is regulated with such precision.

## 2. Results

### 2.1. There are two populations of Cyclin B1 in cells

FCS is an imaging-based technology that relies on fluorescence measurements to estimate a diffusion coefficient. To apply FCS to Cyclin B1 we used CRISPR/Cas9^D10A^ nickase [34] to introduce the mEmerald coding sequence at the 3′ of the CCNB1 open reading frame. Biallelic-tagged clones were identified by PCR and immunoblot analysis (electronic supplementary material, figure S1A). In agreement with previous reports [12,19,35,36], Cyclin B1-mEmerald localized to the cytoplasm, particularly the centrosomes, of interphase cells and was recruited to the spindle, the chromosomes, and the spindle poles of mitotic cells (electronic supplementary material, figure S1B,C). The mitotic timing, spindle assembly checkpoint (SAC) response and chromosome number of the RPE-1 CCNB1-mEmerald^+/+^ cells did not significantly differ from the parental cell line (electronic supplementary material, figure S1D,E), indicating that the addition of mEmerald did not affect Cyclin B1 function and that we could use the fusion protein to report on the proper behaviour of Cyclin B1.

**Figure 1.**
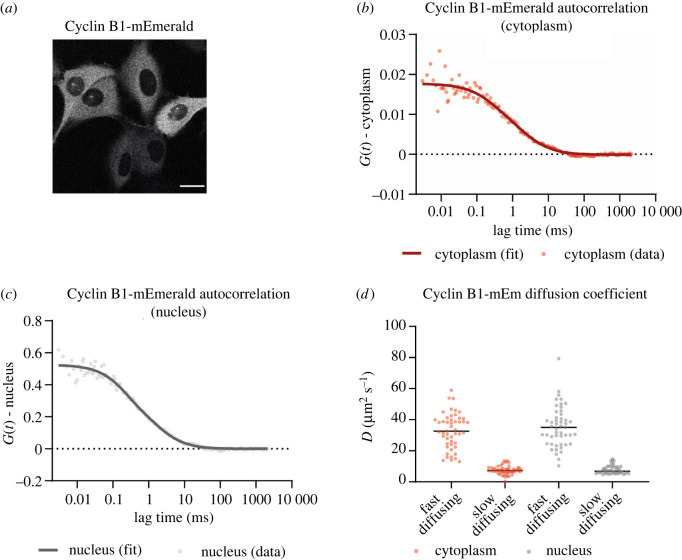
Cyclin B1 size in RPE-1 cells. (*a*) Representative fluorescence confocal image of CCNB1-mEmerald^+/+^ cells. Scale bar corresponds to 20 µm. (*b*) Graph representing the autocorrelation function of Cyclin B1-mEmerald over time in the cytoplasm (*c*) Graph representing the autocorrelation function of Cyclin B1-mEmerald over time in the nucleus (*d*) Dot plot representing the diffusion coefficient of Cyclin B1-mEmerald species. Horizontal black lines represent median values. A total of 49 FCS measurements were obtained in 20 cells in *n* = 3 independent experiments.

The autocorrelation functions (ACFs) from FCS measurements on Cyclin B1-mEmerald fitted better to a 3D two-particle triplet model (3D-2p-triplet) than a 3D one-particle triplet model (3D-1p-triplet) (see Materials and methods), in both the cytoplasm and the nucleus (figure 1*a–c*). This indicated that two populations of Cyclin B1-mEmerald existed: a fast-diffusing fraction with a diffusion coefficient (D) of approximately 35 µm^2^ s^−1^; and a slow-diffusing fraction with a D of approximately 8 µm^2^ s^−1^ (figure 1*d*). The apparent sizes of the two Cyclin B1-mEmerald species can be estimated using the Stokes–Einstein equation (equation (2.1)) provided the viscosity of RPE-1 cells at 37°C is known.

2.1 $$D=\frac{k_{B}T}{6\pi \eta \;r}.$$

To calculate the viscosity of RPE-1 cells, we used RPE-1 cells stably expressing GFP and measured its D in the cytoplasm and nucleus using FCS (electronic supplementary material, figure S2A,B). Fitting the ACFs to a 3D one-particle triplet (3D-1p-triplet) model, we obtained a D of 42 ± 5 µm^2^ s^−1^ for GFP in RPE-1 cells at 37°C. The hydrodynamic radius of GFP was previously reported to be approximately 2 nm [37,38]; therefore, we estimated that the mean viscosity of RPE-1 cells at 37°C was 2.4 ± 0.7 mPa.s in the cytoplasm and 2.6 ± 0.6 mPa.s in the nucleus (electronic supplementary material, figure S2C). We obtained comparable results using the mVenus fluorescent protein (electronic supplementary material, figure S2D). Using these values in equation (2.1) gave the hydrodynamic radius of the fast-diffusing Cyclin B1-mEmerald species as 3–4 nm and the slow-diffusing Cyclin B1-mEmerald species as 8–11 nm. The hydrodynamic radius of Cyclin B1-mEmerald estimated from the structures of the proteins is approximately 4 nm, whereas that of the Cyclin B1-mEmerald-Cdk1-Cks complex is approximately 7.5 nm [3,39]. These values are in the range of the hydrodynamic radii of the two populations that we measured by FCS. Therefore, we conclude that the two populations of Cyclin B1 are an unbound freely diffusing monomer and a fraction of Cyclin B1 bound to Cdk1 and Cks.

**Figure 2.**
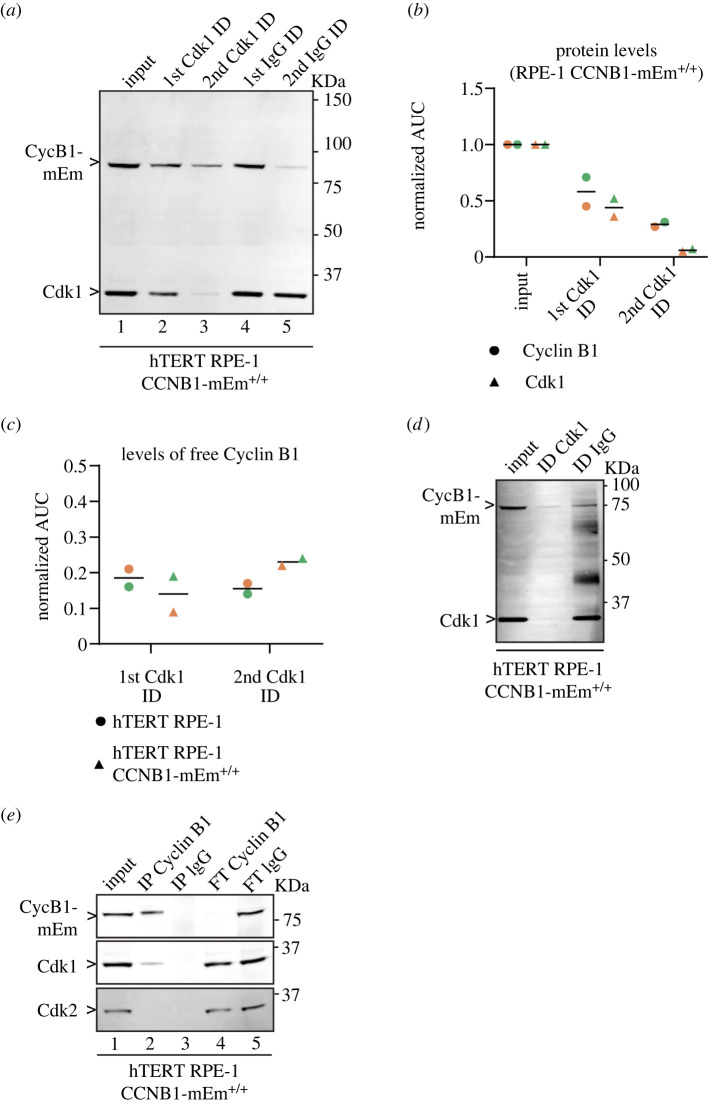
A fraction of Cyclin B1 is not bound to Cdk1 in RPE-1 CCNB1-mEM^+/+^ cells. (*a*) Anti-Cyclin B1 and anti-Cdk1 immunoblot of G2 phase cell lysates before (1st) and after (2nd and 3rd lanes) immunodepleting Cdk1, compared with control immunodepletion with IgG (4th and 5th lanes). (Note that the depletion of of Cyclin B1 on control beads in the 2nd depletion was not consistent, compare electronic supplementary material, figure S3, panels (*a*) and (*c*); we saw depletion in six out of eleven experiments, and neither pre-coating the beads nor low retention tubes solved the problem). (*b*) Quantification of Cyclin B1 and Cdk1 levels before and after immunodepletion of Cdk1. (*c*) Quantification of Cyclin B1 levels before and after immunodepletion of Cdk1 from parental RPE-1 cells and from RPE-1 CCNB1-mEmerald cells. (*d*) Anti-Cyclin B1 and anti-Cdk1 immunoblot of G2 phase cell lysates before and after immunodepletion of Cdk1 to at or below detection levels, or after control immunodepletion. (*e*) Anti-Cyclin B1, anti-Cdk1 and anti-Cdk2 immunoblots of G2 phase cell lysates before (first lane) and after immunoprecipitation with anti-Cyclin B1 antibody second lane), or immunoprecipitation with anti-IgG control antibody (3rd lane) and the unbound fractions from the respective immunodepletions (fourth and fifth lanes). For all graphs, individual dots represent biological replicates, horizontal lines indicate median values. AUC = area under the curve. Molecular mass indicated for all gels on the right. For all panels, *n* = 2 independent experiments. ID = immunodepletion, IP = immunoprecipitation, FT = flow through, CycB1-mEm = Cyclin B1-mEmerald.

To test our conclusion, we used quantitative immunoblotting to assay for Cyclin B1 in cell lysates after immunodepleting Cdk1. Immunodepleting Cdk1 should remove all its bound Cyclin B1 because Cyclin B1 binds Cdk1 with high affinity [3,40]. Two sequential immunodepletions of Cdk1 in G2 phase RPE-1 CCNB1-mEmerald^+/+^ cells reduced Cdk1 to 44.0 ± 11.3% and 6.0 ± 1.4% (Mean ± s.d.) of its original levels (figure 2*a*—compare lane 1 with lane 2 and 3), whereas Cyclin B1 levels dropped to 58.0 ± 18.4% and 29.0 ± 2.8% of its original levels (figure 2*a* – compare lane 1 with lane 2 and 3). This indicated that approximately 18% of Cyclin B1 was not bound to Cdk1 (figure 2*c*). We obtained similar results using parental RPE-1 cells, excluding the possibility that tagging Cyclin B1 affected its binding to Cdk1 (figure 2*c*; electronic supplementary material, figure S3A, B). We observed a Cyclin B1 signal even after depleting Cdk1 below the detection threshold (figure 2*d*; electronic supplementary material, figure S3C), further demonstrating that some Cyclin B1 did not bind Cdk1. (Note that, as expected, we were unable to detect Cdk2 in immunoprecipitates of Cyclin B1 (figure 2*e*; electronic supplementary material, figure S3D—compare lane 2 with lane 1 and 3), indicating that Cdk2 did not bind significantly to Cyclin B1 *in vivo*.) Overall, these data confirmed our conclusions from our FCS measurements that there are two populations of Cyclin B1 in RPE-1 cells: monomeric Cyclin B1 and the Cyclin B1–Cdk1-Cks complex.

### 2.2. Cyclin B1–Cdk1 interaction can be measured using FCCS

Our biochemical data indicated that FCS measurements had accurately identified two populations of Cyclin B1 in living cells; therefore, we should be able to detect the Cyclin B1–Cdk1 interaction *in vivo* using FCCS. With this aim, we generated a RPE-1 cell line expressing a mEmerald-mScarlet fusion protein as a positive control for FCCS, and introduced mScarlet alone into the RPE-1 CCNB1-mEmerald^+/+^ cell line as a negative control [41]. In the cells expressing the mEmerald-mScarlet fusion protein, we obtained an ACF for each fluorophore, plus a cross-correlation function with a cross-correlation quotient *q* of approximately 55–65% (electronic supplementary material, figure S4A). That the cross-correlation quotient *q* was less than 100% is explained by incomplete maturation and extended dark state residence of red fluorescent proteins [42–45]. In the negative control RPE-1 CCNB1-mEmerald^+/+^ cells expressing mScarlet there was no detectable cross-correlation (*q* < 5%) between Cyclin B1-mEmerald and mScarlet (electronic supplementary material, figure S4B).

**Figure 3.**
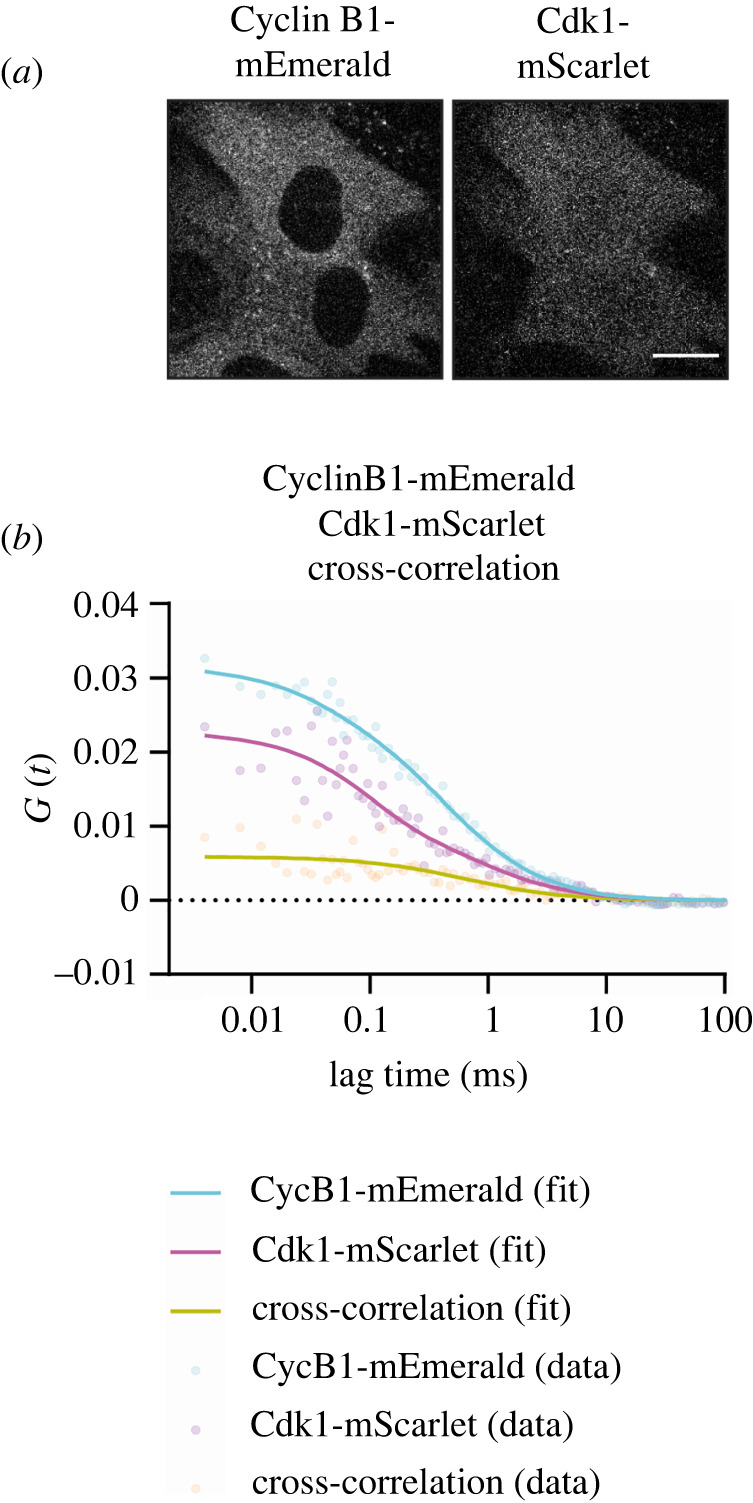
FCCS detects the interaction between Cyclin B1 and Cdk1. (*a*) Representative fluorescence confocal image of RPE-1 CCNB1-mEmerald^+/+^ cells expressing Cdk1-mScarlet from a tetracycline-inducible promoter. Scale bar corresponds to 20 µm. (*b*) Graph of the autocorrelation of Cyclin B1-mEmerald (Cyan), Cdk1-mScarlet (Magenta) and the cross-correlation between the two (Yellow). A total of 44 FCS measurements were obtained in 20 cells in *n* = 3 independent experiments.

Having validated our FCCS measurements, we were in a position to measure the cross-correlation between Cyclin B1 and Cdk1. To enable this, we introduced a tetracycline-inducible construct encoding Cdk1 tagged at the carboxyl terminus with mScarlet (figure 3*a*) into the CCNB1-mEmerald^+/+^ cell line. Co-immunoprecipitation followed by immunoblotting showed that Cyclin B1-mEmerald bound to Cdk1-mScarlet (electronic supplementary material, figure S4C), and FCCS revealed cross-correlation between Cyclin B1-mEmerald and Cdk1-mScarlet (*q* ∼ 25%–35%) (figure 3*b*). We concluded that FCCS could measure protein–protein interactions in living cells.

### 2.3. Time-resolved measurement of Cyclin B1 concentration and complex fraction

**Figure 4.**
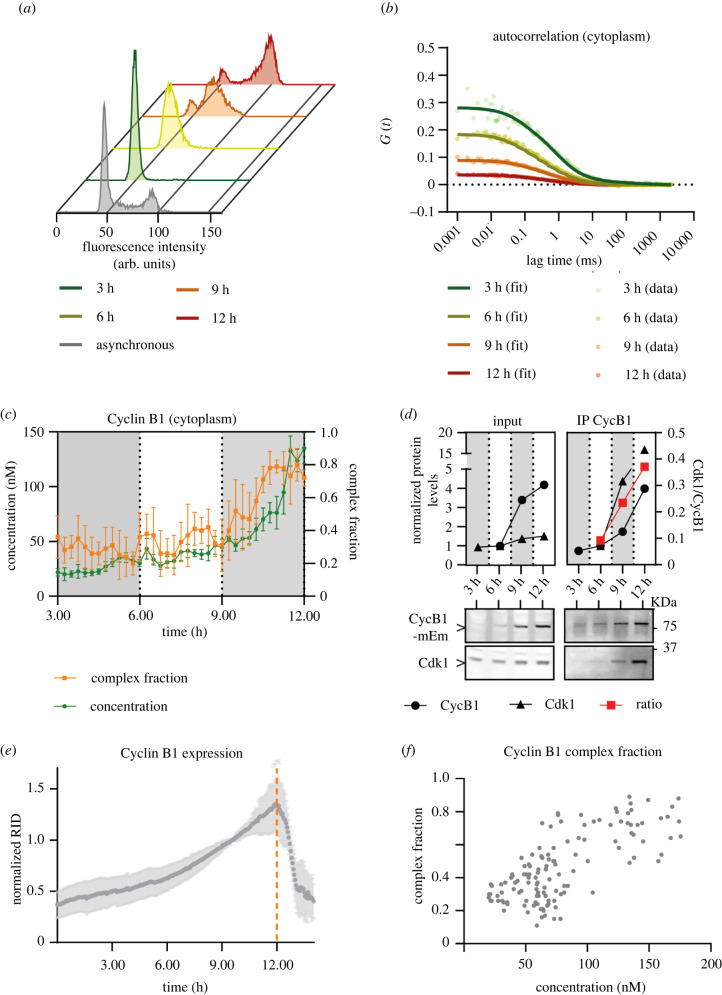
Time-resolved measurement of Cyclin B1 size and concentration. (*a*) Flow cytometry profiles of propidium iodide-stained RPE-1 CCNB1-mEmerald^+/+^ cells: asynchronous and at 3, 6, 9 and 12 h after release from palbociclib arrest. (*b*) Plots of the FCS autocorrelation functions of Cyclin B1-mEmerald at the indicated time points after release from palbociclib arrest. Each dot represents the measurement from one cell. (*c*) Graph showing time-resolved Cyclin B1-mEmerald concentration (left axis) and the fraction of Cyclin B1 in complex with Cdk1 (right axis) estimated from FCS measurements in the cytoplasm. Each point represents the mean of ≥4 measurements from 3 to 4 cells. (*d*) Top panel: quantification of the protein levels measured from anti-Cyclin B1 and anti-Cdk1 immuno-blots (bottom panel, molecular mass indicated on the right) of either synchronized lysates (left) or Cyclin B1 immunoprecipitates (right). The ratio between Cdk1 and Cyclin B1 is plotted in red on the right axis. (*e*) Quantification of Cyclin B1 fluorescence levels (normalized raw integrated density, RID) over time measured by widefield fluorescence microscopy in unsynchronized RPE-1 CCNB1-mEmerald^+/+^ cells. Orange dotted line indicates nuclear envelope breakdown. (*f*) Quantification of FCS-derived measurements of the fraction of Cyclin B1 bound to Cdk1 over total Cyclin B1 levels, plotted against total Cyclin B1 concentration. A total of 125 FCS measurements were obtained in 25 cells in *n* = 3 independent experiments. In all graphs error bars indicate standard deviation. For all panels, *n* = 2 independent experiments unless otherwise specified, time indicates hours after palbociclib release.

Our results revealed that Cyclin B1 existed both as a free monomer and in a complex with Cdk1, and we wondered whether the relative abundance of these two populations changed through the cell cycle. To determine this, we synchronized cells in G1 phase using the Cdk4/6 inhibitor palbociclib [46,47] and assayed cells at specific times after release from the arrest. At each time point, we fixed and stained the cells for flow cytometry-based cell cycle analysis (figure 4*a*), analysed Cyclin B1 size and concentration by FCS (figure 4*b*,*c*; electronic supplementary material, figure S5A,B), and immunoprecipitated Cyclin B1 from cell lysates to assess its binding to Cdk1 (figure 4*d*). FCS measurements revealed that the concentration of Cyclin B1 increased over time following an exponential function (figure 4*c*; electronic supplementary material, figure S5B). We observed a similar Cyclin B1 increase by immunoblot analysis (figure 4*d*—left panel). FCS analysis also revealed that the fast-diffusing monomeric Cyclin B1 was the dominant fraction until 9 h after release, after which the slow-diffusing Cyclin B1–Cdk1-Cks complex became prevalent (figure 4*c*; electronic supplementary material, figure S5B). In agreement with this, we observed an increase in the ratio of Cdk1 binding to Cyclin B1 in Cyclin B1 immunoprecipitates at later time points after release (figure 4*d*—right panel, S5C).

To exclude the possibility that treatment with palbociclib might perturb the normal behaviour of Cyclin B1, we analysed asynchronous cells. The fluorescence level of Cyclin B1-mEmerald increased exponentially before nuclear envelope breakdown (figure 4*e*; electronic supplementary material, figure S5D). This exponential increase measured by widefield epifluorescence agreed with the exponential increase in Cyclin B1-mEmerald concentration in synchronized cells. FCS measurements in asynchronous cells showed a correlation between Cyclin B1 concentration and the fraction of slow-diffusing Cyclin B1 complex in unsynchronized cells: in cells with a low concentration of Cyclin B1 (S phase and early G2 phase), most Cyclin B1 diffused fast, whereas in cells with higher concentrations of Cyclin B1 (mid/late G2 phase) the slowly diffusing Cyclin B1 population was dominant (figure 4*f*). This agreed with the data in figure 4*c*. Thus, we concluded that synchronization with palbociclib did not perturb the behaviour of Cyclin B1, and that the percentage of Cyclin B1 bound to Cdk1 changed from about 30–40% in S phase to about 70–80% in late G2 phase.

### 2.4. Estimating Cyclin B1–Cdk1 binding affinity in living cells

We wanted to know whether the change in the proportion of Cyclin B1 binding to Cdk1 as cells progressed through G2 phase would fit with a simple model where binding to a constant amount of Cdk1 was driven by an increase in concentration of Cyclin B1. We modelled Cyclin B1 expression with a single exponential function, and fitted Cdk1 to a straight-line equation, using 629 nM as the initial Cdk1 concentration (inferred from [48]) and 28 nM as the Cyclin B1–Cdk1 *K_D_* (dissociation constant [3]). We calculated the fraction of Cyclin B1 in complex with Cdk1 according to our model using equation (2.2)

2.2 $$K_{D}=\frac{\left[\mathrm{Cdk}1^{\mathrm{Free}}][\mathrm{CycB}1^{\mathrm{Free}}\right]}{\left[\mathrm{CycB}-\mathrm{Cdk}1\right]}.$$

**Figure 5.**
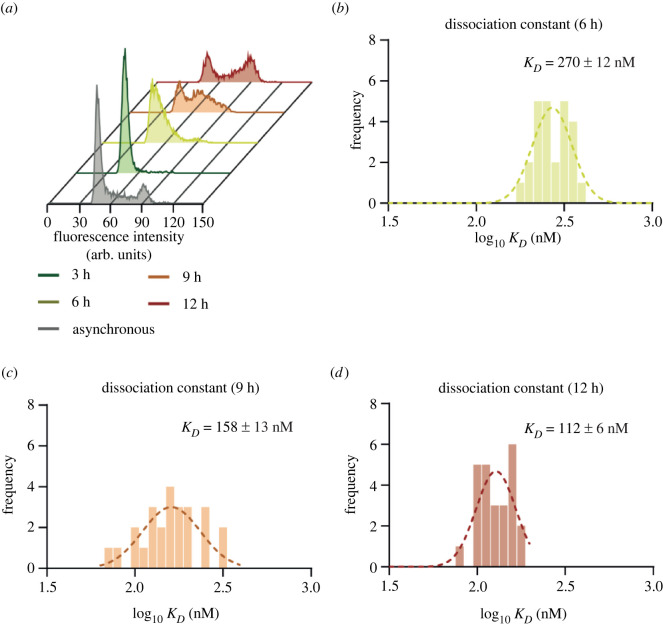
The dissociation constant of the Cyclin B1–Cdk1 complex decreases through the cell cycle. (*a*) Flow cytometry profiles of propidium iodide-stained RPE-1 CCNB1-mEmerald^+/+^ Cdk1-mScarlet cells: asynchronous and at 3, 6, 9 and 12 h after release from palbociclib-arrest. (*b–d*) Frequency histograms of the log (*K_D_*) values measured for Cyclin B1-mEmerald and Cdk1-mScarlet by FCCS (see 'Material and methods') at 6 (*b*), 9 (*c*) and 12 (*d*) hours after release from a palbociclib-arrest. In all histograms, the dotted line represents the Gaussian fit. *K_D_* is indicated as mean ± s.e.m. (standard error of the mean). Each histogram is a plot of *K_D_* derived using 40–45 FCCS measurements across four to five cells per experiment. For all panels, *n* = 2 independent experiments.

Using these parameters, more than 95% of Cyclin B should have been bound to Cdk1 even at low levels of Cyclin B1, which did not match our experimental data (figure 4*c*). The discrepancy could be because the value of the dissociation constant (*K_D_*) measured *in vitro* might be different *in vivo*, where the conformation and interactions of proteins might vary considerably (reviewed in [49]). This prompted us to use FCCS to measure the *K_D_* of the Cyclin B1–Cdk1 complex *in vivo* (see 'Material and methods'). We arrested RPE-1 CCNB1-mEmerald^+/+^ cells expressing Cdk1-mScarlet in G1 phase with palbociclib and measured the *K_D_* for Cyclin B1–Cdk1 at different time points following release from the arrest. The synchrony of the cells was assayed in parallel using flow cytometry (figure 5*a*). We measured the *K_D_* for cells at 6, 9 and 12 h after release (the low levels of Cyclin B1 prevented us from measuring the *K_D_* at 3 h). We observed the effective K_D_ for Cyclin B1-mEmerald and Cdk1-mScarlet reduced from 270 nM in early G2 phase (6 h post release from palbociclib) to 112 nM just before mitosis (12 h post release from palbociclib; figure 5*b–d*). Using 112 nM as the *K_D_*, our model (equation (2.2)) predicted that 87% of Cyclin B1 should be in complex with Cdk1 at 12 h post palbociclib release, in better agreement with the 79% obtained through FCS.

Altogether, these results demonstrate that the *K_D_* value of Cyclin B1–Cdk1 interaction was higher *in vivo* (112 nM) than *in vitro* (28 nM), and that the affinity between Cyclin B1 and Cdk1 increased as cells progressed through G2 phase to peak just before mitosis. This implied that the binding between Cyclin B1 and Cdk1 might be a regulated step.

## 3. Discussion

To understand how the multiple components of the cell cycle machinery coordinate the profound changes in cell architecture with temporal and spatial precision requires that we can measure the assembly and disassembly of regulatory complexes in proliferating cells. FCS and FCCS provide quantitative information on living single cells that combines some of the advantages of biochemistry with those of imaging, such that we can measure protein concentration and protein complex assembly and disassembly in a spatially and temporally defined manner, making them valuable tools to study the rapid events underlying cell cycle progression.

FCS and FCCS have been previously used as tools to study cell cycle; in particular, pioneering work using high-throughput FCS by the Ellenberg lab [50,51]. Wachsmuth and colleagues measured the temporal changes in diffusion, concentration and cross-correlation ratio of the cell cycle proteins Aurora B and INCENP (Inner Centromere Protein) [50]. Walther *et al*. [51] used FCS of endogenously tagged condensins to measure the number of condensin complexes on mitotic chromosomes [51]. In our study, we additionally measured intracellular viscosity in order to estimate the hydrodynamic radii of Cyclin B1 and Cyclin B1–Cdk1 complex. This represents a technical advance because it is very difficult to separate complexes with such similar sizes by biochemical methods such as size-exclusion chromatography. Furthermore, we used FCCS to measure *in vivo* the binding affinity between Cyclin B1 and Cdk1.

The pattern of Cyclin B1 expression we observed (figure 4) is in line with previous studies on Cyclin B1 promoter activity in different cell lines and with different synchronization strategies [17,52–54], but there is some discrepancy regarding the concentration of Cyclin B1. We estimate that in RPE-1 cells the concentration of Cyclin B1 increases from approximately 20 nM in S phase to approximately 150 nM in late G2 phase. Our measurements agree with previous studies [48,55,56], but other works reported higher concentrations [57–59]. The variability is likely explained by differences in the cell lines measured and in the methodology. Indeed, FCS tends to underestimate concentrations due to the complex photophysics, incomplete maturation and fluorescence probability (p_f_) of fluorescent proteins—for example, green fluorescent proteins have a p_f_ of approximately 70–80% and red fluorescent proteins have a p_f_ of approximately 50–60% [42–45,60]. On the other hand, immunoblotting-based measurements are complicated by the requirement for careful calibration of the antibodies and a linear detection method. Although Cdk1 levels are often considered to be constant during the cell cycle, our quantitative immunoblotting revealed that Cdk1 levels increase by 60% as cell progress through G2 phase (figure 4*d*), in agreement with several other reports [61–63]. It is not clear whether newly synthesized Cdk1 has different properties compared to the Cdk1 persisting from the previous cell cycle, but if it does this could offer an explanation for the regulated assembly of Cyclin B1–Cdk1 complexes that our data imply (see below, [64]).

Our FCS measurements reveal that Cyclin B1 exists as two distinct species in RPE-1 cells during interphase: monomeric Cyclin B1, and Cyclin B1 in complex with its interacting partner Cdk1 (figure 1). We validated this result using Cdk1 immunodepletion (figure 2) and FCCS with Cdk1-mScarlet (figure 3). (Note that the non-specific depletion of Cyclin B1 with the control IgG does not change this conclusion since the effect would be to underestimate the amount of free Cyclin B1.) A pool of monomeric Cyclin B1 is in line with previous conclusions from cell lysates [17,57] but to our knowledge our study represents the first measurement of such a pool in intact cells. Using FCS in a time-resolved manner, we observed a sharp increase in the fraction of Cyclin B1 binding to Cdk1 in late G2 phase (figure 4), which correlates with an increase in affinity between the two proteins (figure 5). The effective *K*_D_ values we estimate from FCCS are higher than those reported *in vitro* [3,40] but this can be explained by (a) the molecular crowding of the cytoplasm compared to the environment found in a test tube; (b) the photophysics of the fluorophores influencing FCCS measurements; (c) the competition for Cyclin B1 between endogenous Cdk1 and Cdk1-mScarlet [44]; and (d) the competition of Cdk1 for binding partners other than Cyclin B1, for example Cyclin A2 (although by FCS we measured the concentration of Cyclin A2 in the cytoplasm of G2 cells as only 40 ± 14 nM, data not shown). Nevertheless, the increase in affinity between Cyclin B1 and Cdk1 as cells progress through the cell cycle implies some regulation of the binding dynamics of the two proteins. The nature of this regulation is as yet undefined, but it is likely to be one or more post-translational modifications. A potential mechanistic explanation is through modulation of the phosphorylation of Cdk1 on its T-loop (T161), which favours Cdk1-Cyclin B1 binding [65–69]. T161 is phosphorylated by CAK (Cdk1 activating kinase), however the major CAK in vertebrate cells—a trimeric protein complex comprised of Cdk7, Cyclin H and MAT1 (Ménage à trois 1)—is constitutively active during the cell cycle [67,70–75]. Thus, the change in K_D_ between Cyclin B1 and Cdk1 may be due to a limiting amount of Cdk7 activity, which *in vivo* is required to stabilize other complexes, for example Cdk1-Cyclin A. In *Drosophila*, Cdk7 is sequestered in the cytoplasm until prophase, possibly reflecting a need for increased CDK activity later in mitosis [76]. Conversely, the phosphatases that remove T-loop phosphorylation could be regulated. The T-loop of monomeric Cdk2 is dephosphorylated by phosphatase 2C and CDKN3 (Cyclin-dependent kinase inhibitor 3; formerly known as KAP, kinase-associated phosphatase) [77–80], but their role in regulating Cdk1 and cell cycle regulation is less clear [81,82]. In this context it is worth notice that even in the active Cyclin B1–Cdk1 complex the phosphorylated T-loop of Cdk1 remains accessible to solvent and therefore to phosphatase activity [3]. Alternatively, Coulonval and colleagues reported coupling between the phosphorylation of T14 and T161 on Cdk1, whereby T14 phosphorylation influenced T161 phosphorylation and interaction with Cyclin B1 [83]. Aside from phosphorylation, K-33 acetylation also appears to affect the interaction between Cdk1 and Cyclin B1, and may be subject to cell cycle regulation [84]. Although our study identified monomeric Cyclin B1 and Cyclin B1 bound to Cdk1 as the two main forms of Cyclin B1 in interphase RPE-1 cells, this situation may change during mitosis, as Cyclin B1 can form stable complexes with other proteins including MAD-1 [10–12], separase [13–15] and the anaphase promoting complex/cyclosome [85,86].

In conclusion, we have established the means to measure the kinetics with which protein complexes assemble and disassemble in living cells, and our data reveal a previously unsuspected regulated step in the entry to mitosis where the assembly of the major mitotic kinase, Cyclin B1 and Cdk1, increases as cells progress through G2 phase.

## 4. Material and methods

### 4.1. Cell culture and synchronization

hTERT RPE-1 FRT/TO cells were cultured in F12/DMEM (Sigma-Aldrich) medium supplemented with GlutaMAX (Invitrogen), 10% FBS (Gibco), 0.348% sodium bicarbonate, penicillin (100 U ml^−1^), streptomycin (100 µg ml^−1^) and Fungizone (0.5 µg ml^−1^). Cells were maintained in a humidified incubator at 37°C and 5% CO_2_ concentration. For live-cell imaging experiments cells were imaged in Leibovitz L-15 (Thermofisher) medium supplemented with 10% FBS, penicillin (100 U ml^−1^) and streptomycin (100 µg ml^−1^).

G2-synchronization was achieved through a 24-h treatment with 100 nM palbociclib (Selleckchem) followed by 12 h release into normal medium, as described in [46,47].

Where indicated, cells were stained with 20 nM sirDNA (Spirochrome) following manufacture's protocol, treated with 100 nM Taxol (Sigma-Aldrich) or 55 nM nocodazole (Sigma-Aldrich).

### 4.2. Gene editing

For CCNB1 tagging, RPE-1 FRT/TO cells were transfected using 500 ng of a modified version of the PX466 ‘All-in-One' plasmid containing Cas9D10A-T2A-mRuby and gRNAs targeting CCNB1 (5′-ACCGTTTACTTTTAATAAAGCTTG-3′ and 5′-ACCGTAATATGTACAGATGGCACA-3′). The all-in-one plasmid was cotransfected with 500 ng of repair plasmid designed as a fusion of LINKER-mEmerald flanked by two 850 bp arms, homologous to the genomic region around the Cas9 cutting site. 72 h post transfection, 50 000 mRuby positive cells were sorted in a 1 cm well and expanded for one week before a second sorting of single cells in 96 well plates. The presence of mEmerald tag was identified through PCR using primers forward 5′-CAAATGCTTCTCCTATGTGACAGG-3′ and the reverse 5′-TTCAGGTGGGTGGGATTTAG-3′. PCR products of positive clones were sequenced using the same primers.

For Cdk1-mScarlet expression, RPE-1 FRT/TO CCNB1^+/+^ cell line was transfected with pcDNA5-FRT/TO-Cdk1-alpha-mScarlet and pOG44 (Invitrogen) using a 1 : 5 ratio. For mEm-alpha-mScarlet and mEmerald and mScarlet expression, RPE-1 FRT/TO cells were transfected with either pcDNA5-FRT/TO-mEmerald-alpha-mScarlet or pcDNA5-FRT/TO-mScarlet together with pOG44 using a 1 : 5 ratio. All transfections were followed by a two weeks selection using Geneticin (Gibco) 0.4 mg ml^−1^. Gene expression was induced using tetracycline (Calbiochem) 1 µg ml^−1^. In FCS experiments tetracycline was added 3 h before imaging, in immunoprecipitation (electronic supplementary material, figure S4C) tetracycline was added 16 h before lysis.

All transfections were performed by electroporation using a Neon Transfection System (Invitrogen) with two pulses at 1400 V for 20 ms, using 1 µg total DNA per million cells.

### 4.3. Protein extraction

In electronic supplementary material, figure S1, RPE-1 cells were trypsinized and incubated in lysis buffer (150 mM NaCl, 50 mM Tris pH 7.4, 0.5% NP-40) supplemented with HALT protease/phosphatase inhibitor cocktail (Thermo Fisher Scientific) for 30 min at 4°C before clarification.

In figures 2 and 4, and electronic supplementary material, figure S4, following G2-Synchronization, RPE-1 cells were trypsinized and resuspended in IP buffer (150 mM NaCl, 50 mM Tris pH 7.4, 2.5 mM MgCl2, 1 mM EGTA, 1 mM DTT, 1 mM PMSF) supplemented with HALT protease/phosphatase inhibitor cocktail. Cells were lysed through N_2_ cavitation, incubating the cells at 1500 psi, 20 min at 4°C and rapidly releasing the pressure.

In all experiments, lysates were then clarified through centrifugation (14′000*g*, 20 min, 4°C) and then quantified using Bradford Reagent (Bio-Rad Laboratories) according to manufacturer's instructions.

### 4.4. Immunodepletion and immunoprecipitation

For Cdk1 immunodepletion (figure 2; electronic supplementary material, figure S3), 100 µg of clarified lysates were diluted to a final concentration of 2 µg µl^−1^ and incubated with 60 µl of Dynabeads prebound to 7.5 µg of either anti-Cdk1 Antibody (BD Biosciences, 610 037) or Mouse Ig-G, in a total volume of 100 ul for 2 h at 4°C. The incubation with beads was repeated two (figure 2*a*; electronic supplementary material, figure S3A) or three times (figure 2*d*, electronic supplementary material, figure S3D), depending on the experiment. Forty micrograms of either the immunodepleted lysates or the input were used for SDS-PAGE.

For Cyclin B1 immunoprecipitation (figure 2*e*; figure 4*d*; electronic supplementary material, figure S3d), 30 µl of Dynabeads were cross-linked to 3.75 µg of either anti-Cyclin B1 antibody (GSN1; SantaCruz, sc-245) or Mouse Ig-G, through incubation in 20 mM dimethyl pimelimidate solution, 20 min, RT. 200 ug of clarified lysates were then added to the beads and incubated for 3 h at 4°C.

For mEmerald and mScarlet immunoprecipitation (electronic supplementary material, figure S4C), 30 µl of either GFP-Trap, RFP-Trap (Chromotek) or cross-linked IgG control (see above) magnetic beads were incubated 2 h at 4°C with 400 ug of clarified lysates diluted to a final concentration of 2 µg µl^−1^.

For all IPs, beads were washed five times with IP buffer and then incubated 5 min at 65°C in 30 µl 2X Sample Loading Buffer, prior to SDS-PAGE.

### 4.5. Immunoblotting

Forty micrograms of RPE-1 cell lysates were separated through SDS-PAGE on a 4–12% NuPAGE gel (Invitrogen) and transferred to an Immobilon-FL polyvinylidene fluoride membrane (IPFL00010, Millipore). The membrane was blocked with 5% Milk, 0.1% Tween, PBS and incubated overnight with primary antibodies at 4°C in 2.5% Milk, 0.1% Tween, PBS. The following day the membrane was washed with 0.1% Tween PBS and incubated with secondary antibodies in 2.5% Milk, 0.1% Tween, PBS for 1 h at RT.

Primary antibodies were used at the indicated concentrations: and anti-CCNB1 (1 : 1000, SantaCruz, GSN1, sc-245), anti-Cdk1 (1 : 1000, BD Biosciences, 610 037), anti-Cdk2 (1 : 1000, 78B2, Cell Signalling), anti-Tubulin (1 : 3000, ab6046, Abcam). IRDye800CW donkey anti-mouse (926-32212, LI-COR), IRDye800CW donkey anti-rabbit (926-32213, LI-COR), IRDye680CW donkey anti-mouse (926-68072, LI-COR), and IRDye680CW donkey anti-rabbit (926–68073, LI-COR) secondary antibodies were all used at 1 : 10 000.

Proteins were visualized with LI-COR Odyssey CLx scanner (LI-COR Biosciences). Western blot quantification in figure 2 and electronic supplementary material, figures S3 and 4D, was performed calculating the area under the curve using Fiji's ‘Gel' plugin. Values were adjusted by fitting to a straight-line function obtained by immunoblotting serial dilutions of protein lysate. In figure 2 and electronic supplementary material, figure S3, values were normalized to the input value. Regarding figure 4*d*, values were normalized on the 6 h time point.

### 4.6. Live-cell imaging

Mitotic time measurements (electronic supplementary material, figure S1D) were obtained using differential interference contrast (DIC) imaging on a Nikon Eclipse microscope (Nikon) equipped with a 20 × 0.75 NA objective (Nikon), a Flash 4.0 CMOS camera (Hamamatsu) and an analyser in the emission wheel for DIC imaging. Single plane images were taken every 3 min, for 24 h using micromanager software (µManager) and analysed using FiJi (ImageJ).

Image series displayed in electronic supplementary material, figure S1B and C were obtained on a Marianas confocal spinning-disk microscope system (Intelligent Imaging Innovations, Inc.) equipped with a laser stack for 445 nm/488 nm/514 nm/561 nm lasers, a 63 × 1.2 NA objective (Carl Zeiss) and a Flash4 CMOS camera (Hamamatsu). 8 Z stacks (Step size = 1 µm) were taken every 30 s, for 90 min, using 20% 488 nm laser power and 10% 647 nm laser power, for 50 ms exposure, using Slidebook 6 software (Intelligent Imaging Innovation, Inc.).

Widefield microscopy experiments (figure 4*f*; electronic supplementary material, figure S5D) were performed using an Nikon Eclipse microscope (Nikon) equipped with a 40 × 1.30 NA objective (Nikon) and a Flash 4.0 CMOS camera (Hamamatsu), recording 488 nm emission with 150 ms exposure. Single plane images were taken every 3 min, for 24 h using micromanager software (µManager) and analysed using FiJi (ImageJ). Raw Integrated Density (RID) of the whole cell was normalized to the RID of the same cell 50 frames (150 min) before nuclear envelope breakdown. Multiple asynchronous cells were aligned on their NEBD time.

### 4.7. Chromosome spreads

For chromosome spreads (electronic supplementary material, figure S2 E,D), following a 3-hours treatment with 100 ng ml^−1^ colcemid (GIBCO) cells were trypsinized and recovered in a falcon tube. Cell suspension was centrifuged for 3 min at 250 g and resuspended in 5 ml of 75 mM KCl, added dropwise. After a 15 minute-incubation at 37°C, 10 drops of Carnoys Fixative (3 : 1 methanol : acetic acid) were added. Following a 5 min centrifugation at 200 g, the cell pellet was resuspended in 5 ml of Carnoys Fixative. After 90 min at −20°C, a second fixation was performed using 5 ml of Carnoys Fixative at room temperature for 15 min. Cells were then centrifuged at 200 g for 5 min, the supernatant removed, and the pellet resuspended in 200 µl of Carnoys Fixative. Spreads were performed by dropping the cells on wet slides in a wet chamber from 30 to 40 cm height. Chromosome spreads were aged at room temperature for 30 min, then incubated for 30 min with 1 : 50 Giemsa stain : Giemsa buffer, and finally washed in PBS pH 7.8. Once dried, slides were mounted with DPX mountant (Sigma-Aldrich) and coverslips and incubated overnight at room temperature.

Transmitted light images of metaphase spreads were captured using a 63 × 1.4 NA lens on a Marianas confocal spinning-disc microscope system, and the number of chromosomes per cell was counted using ImageJ software.

### 4.8. Flow cytometry

In flow cytometry experiments (figures 4*a* and 5*a*), cells were detached, washed with PBS and fixed with 70% ethanol 4 h –20°C. After fixation cells were stained 20 min in a 1 µg ml^−1^ propidium iodide (PI) solution, supplemented with 10 µg ml^−1^ RNAse (Sigma). Stained cells were acquired using a LSR II flow cytometers (BD Bioscience). Cell cycle profiles were analysed using the software FlowJo.

### 4.9. Calculating Cdk1-CycB binding

To calculate the fraction of Cyclin B1 engaged in Cdk1 binding over time, Cyclin B1 concentration increase was modelled by fitting FCS calculation (figure 4*c*) with a single exponential function (equation (4.1)).

4.1 $$y=y_{0}e^{k*x}$$

where *y*_0_ = 5.561 and *k* = 0.04232, *R* = 0.8703. Cdk1 increase was modelled using a straight-line equation based on figure 4*d* and using initial Cdk1 concentration of 629 nM [48], slope = 0.1509, *y*_0_ = 74.37, *R* = 0.9292.

### 4.10. Data analysis and statistics

Statistical analysis, fitting and plotting were performed with Prism 8 (GraphPad). Graphs in electronic supplementary material, figure S2 were realized using following the ‘Superplots' pipeline and Python 3.7.0 [87].

### 4.11. FCS instrumentation and measurements

The FCS and FCCS experiments were performed on a Leica TCS SP8 confocal microscope (DMI8; Leica). The samples were illuminated using a white light wavelength-adjustable pulsed laser that was focused to the back focal plane of a Leica HC PL APO CS2 63x/1.20 water immersion objective. The wavelengths used were 488 nm for samples expressing mEmerald or EGFP, and 569 nm for samples expressing mScarlet. The pinhole size was set to 1 airy unit and the emitted signal was recorded using Leica HyD SMD (single molecule detection) detectors with user-adjustable detection range. For mEmerald and EGFP, we used a detection range of 505–540 nm and for mScarlet a detection range of 580–625 nm.

Prior to each FCS/FCCS experiment, the objective's collar was corrected to reduce aberrations, and the system was calibrated using Atto 488 and Atto 565 to determine the effective confocal volume and structure factor at 488 nm and 569 nm excitation, respectively. The cells seeded on a µ-Slide 8 well ibiTreat dish were then measured for 10 s at 37°C. The recorded signal was computed to generate auto- and cross-correlation functions and fit using Leica LAS X SMD FCS module. All measurements were fit with a three-dimensional free diffusion triplet (3D-triplet) model. The number of diffusing components in the fitting model were determined using Akaike information criterion (AIC) and F-tests in GraphPad Prism [88]. We found that for CCNB1-mEm, a 3D two-component triplet model (3D-2particle-triplet model) is the most suitable model, whereas for freely diffusing EGFP in RPE-1 cells, 3D one-component triplet model (3D-1particle-triplet model) is the more appropriate model.

In our FCCS experiments, we calculated the cross-correlation quotient *q* as the ratio of the CCF amplitude to the ACF amplitude. The *q* value is a measure of the amount of cross-correlation between the two species which is a representative of the fraction of molecules in complexes [89]. The dissociation constant (*K_D_*) for the interaction between Cyclin B1-mEmerald and Cdk1-mScarlet was calculated using equation (2.2). The concentrations of unbound Cyclin B1-mEmerald, unbound Cdk1-mScarlet and Cyclin B1-mEmerald-Cdk1-mScarlet complex for calculating *K_D_* were estimated using FCCS as detailed in [44,90].

## Data Availability

The data are provided in the electronic supplementary material [91].
